# Absence of laws regarding sperm and oocyte donation in Japan and the impacts on donors, parents, and the people born as a result

**DOI:** 10.1002/rmb2.12329

**Published:** 2020-06-07

**Authors:** Yuri Hibino, Sonia Allan

**Affiliations:** ^1^ Department of Environmental and Preventive Medicine Graduate school of Medical Science Kanazawa University Kanazawa Japan; ^2^ School of Law Western Sydney University (from 1 July 2020) Sydney NSW Australia; ^3^ Griffith Law School Griffith University Mount Gravatt Qld Australia

**Keywords:** children's rights, egg donation, Japan, law, sperm donation

## Abstract

An absence of any statutory law in Japan regarding donor conception creates uncertainty about the status of donors in relation to the child(ren) born as a result. Laws that provide for certainty regarding the status of the donor are called for, as are laws that address donor anonymity. It would be pragmatic to introduce a prospective system that requires open donation, allowing information to be recorded and released to donor‐conceived people upon request. For past donations, a voluntary register should be established, which would allow those people who are seeking information to register this.

## 1 INTRODUCTION

Despite assisted reproductive treatment (ART) using artificial insemination with donor sperm having been reported as early as 1949 in Japan and oocyte donation occurring more recently, there is no statutory law regulating ART or third‐party reproduction. Attempts at passing legislation have failed, while academic society guidelines and government documents have been functioning as a soft law. In the meantime, the practice of donor conception using donor sperm and oocytes has proceeded both in country and, in the latter case, using cross‐border reproduction. This has left the status of donors in relation to the children born as a result unclear, and the rights of people born via donor conception to access information about their genetic heritage unsupported. In this paper, the authors outline current situation in Japan regarding donor conception and recommendations are made to improve current and future practices and regulation.

## 2 ARTIFICIAL INSEMINATION WITH DONOR SPERM

Artificial insemination with donor sperm (AID) has been practiced for many years in Japan to assist heterosexual couples to have a child, with an emphasis on donor anonymity. The total number of births through AID with anonymous sperm donation is not known exactly—some reports varying from more than 10 000 to more than 15 000 individuals. The principle donors have been reported to have been medical students.

Keio University Hospital has long been a mainstay in offering anonymous sperm donation for married heterosexual couples since 1949. Since 1995 when Dr Yasunori Yoshimura became a professor at Keio University Hospital (retired in 2014), donor records have been kept rigorously, although still anonymized and never intended for disclosure. Before him, records may have been destroyed.

In September 2018, it was reported in the news media that Keio University Hospital had stopped receiving new patients seeking donor sperm. The hospital reported that fewer donors were willing to participate once they were made aware of the spreading global recognition of children's rights to know their biological parents.^1^ However, a reduction in donors may be confounded by a lack of laws that clarify the legal status of the donor. For example, this may create fear that exposing a donor's identity may lead to inheritance claims by donor‐conceived individuals. It is also worth noting that despite the reduction in donors at the central hospital, private sperm donation has been reported to be increasing and flourishing.^2^

Private donation in Japan can be provided via organizations or individuals including that in some cases, sperm is delivered by donors to potential parents face to face. In these cases, the donor is only semi‐anonymous. The academic/medical establishment has warned that private sperm donation can be dangerous with respect to legal heritage and infectious diseases.

## 3 OOCYTE DONATION IN JAPAN (AND CROSS‐BORDER REPRODUCTION)

Demand for oocyte donation has increased over the years, as women are marrying later and age‐related infertility increases. There are again no statutory laws governing oocyte donation in Japan. Government reports maintain that commercial oocyte donation is not permitted, while non‐commercial, anonymous oocyte donation in principle is allowed.

Egg donation has been conducted publicly by two organizations. The Japanese Institution for Standardizing Assisted Reproductive Technology (JISART) established in 2003 reported that 77 patients were treated at JISART clinics and 38 children born between 2007 and 2017. Their guidelines allow donated egg from known donor such as relatives. The Oocyte Donation Network (OD‐Network), which was established in 2013,^3^ reported that the first child was born as a result of oocytes from anonymous volunteer egg donor sourced from the OD‐Network in 2017.^4^

As the domestic pathway for oocyte donation has continued to be extremely limited, some couples have sought overseas oocyte donation programs provided by Japanese agencies. Such arrangements are most often commercial and anonymous. While some travel to the United States, others preferred Thailand due to lower pricing and lax laws. Donor records may have been kept by the clinic or Japanese agent but, as the practice was clandestine, the records are vulnerable unlikely to be kept for many years.

When commercialization was prohibited in Thailand in 2015,^5^ Taiwan became a popular destination for Japanese couples seeking oocyte donors. Taiwan has been seen to have advantages in relation to there being a Japanese population that can provide interpreters, geographical proximity, and a pro‐Japanese culture. Cross‐border reproduction has generally been welcomed, according to Ministry of Health and Welfare Taiwan, with the numbers of egg donation cycles reported to be 316 in 2010, 508 in 2012, and 836 in 2014 (noting that such statistics are not limited to those coming from Japan).

In Taiwan, remuneration for gamete donation has been legal since the Artificial Reproduction Act was enacted in 2007.^6^ Under that law, donors are anonymous, but donor‐conceived individuals can ask public officials before marriage whether intermarriage (marriage to a relative) will happen. Donor privacy is completely protected, and identifiable information is never disclosed.

## 4 THE STATUS OF THE DONOR

For both AID and oocyte donation, there are no laws in Japan that specifically govern the parent‐child relationships resulting from ART and/or donor conception procedures. However, pursuant to Article 772, para.1 of the Civil Code (Presumption of Child in Wedlock) “a child conceived by a wife during marriage shall be presumed to be a child of her husband,” this would mean that the child born as a result of AID to a married couple would be considered the legal child of the husband. However, note, the presumption is rebuttable, and the status of the donor in such circumstances is not explicitly provided for. The status of the donor in relation to a child born to a single woman or woman in a same‐sex couple who has used donor sperm to conceive that child is unclear.

In relation to oocyte donors, the Japanese Civil Code does not address whether the recipient woman, who will also be the birth mother, is the legal mother. However, in a case concerning cross‐border surrogacy and the status of a child born as a result, the Supreme Court of Japan in 2006 held that a woman who had delivered a child (which means surrogate mother) is legal mother of a child. Presumably, this reasoning would also apply to the recipient mother of donated oocytes who gives birth to the child in circumstances that do not involve a surrogacy arrangement. This should be confirmed by legislature to avoid any doubt.

It is clear that without specific legislation that clarifies the status of the donor and the recipient parents within the context of donor conception, the rights and/or responsibilities of all involved are uncertain.

## 5 ABOLITION OF ANONYMITY

Over the last several decades, the regulation of ART, including donor conception, has been drastically changing across the world. In relation to donor anonymity, it appears that a transition to openness and non‐anonymity of donors is growing.^7^, ^8^, ^9^, ^10^, ^11^ This includes legislation in the Australian states of New South Wales, Victoria, Western Australia, and the countries of Austria, Argentina, Croatia, Finland, Ireland, the Netherlands, New Zealand, Norway, Sweden, Switzerland, the United Kingdom and Uruguay.

In the Australian state of Victoria, legislation came into force in March 2017, which granted *all* donor‐conceived individuals the opportunity to receive identifying information about past donors regardless of when the donation took place.^12^, ^13^, ^14^, ^15^ The model adopted in Victoria is a world first in that it mirrored the approach taken in Australian states (such as NSW) in the 1980s that enabled adoptees to access *information* about their genetic/biological origin, regardless of when they were born, while providing for a person to file a “contact veto” if they did not want to be *contacted*.^16^, ^17^ Similarly, donors and donor‐conceived individuals were given the right to submit contact preferences, which do not prevent the release of information, but do allow individuals to state whether, and if so, how they wish to be contacted by others. Such legislation resolved inconsistencies in previous law that granted some donor‐conceived people access to information but denied others such access based on when the donation took place, while balancing the right to privacy for donors who donated under a regime of anonymity.

Interestingly, the Swiss laws also allowed for retrospective release of information in 2004, but it is the more recent Victorian laws that have been reported in the news media to have influenced some practices in Japan (such as the abovementioned media report that one university hospital stopped accepting new patients due to fears of retrospective abolition of donor anonymity).^1^

At the same time, the situation regarding donor anonymity is changing drastically due to the existence of direct‐consumer DNA testing.^18^, ^19^ People can access their genealogies, and, in some cases, donor‐conceived people can find their donor or donor siblings through DNA database searches. This means that regardless of laws or practices that support donor anonymity, donor‐conceived people may gain direct access to the information they seek. It is therefore important to consider what the implications are for Japan.

## 6 RECOMMENDATIONS

Legislation is called for which must be culturally sensitive, while also recognizing that where donor conception is practiced, there is an increasing likelihood that a person will learn of their donor conception status, and some may seek information about their genetic heritage and relatives. At the same time, it is important to consider that Japan's society is conservative. Much stigma remains around infertility and quite possibly for donors who donated sperm in the past. It may not be appropriate, in the first instance to introduce retrospective legislation that reveals the identity of past donors. Instead, it would be a pragmatic approach to introduce a prospective system that implements an open system of donation that is no longer anonymous. This would seem indicated given the modern social environment for donor conception appears to have moved toward openness since private sperm donation has been increasing recently in Japan.

For past donations, it would likely be more socially acceptable to implement a voluntary register, which would allow those people who are seeking information to register their desire to know. It is suggested here that such a register should involve active outreach to donors, should a donor‐conceived person seek information. Note, although it is reported that one‐third of past donor information is missing,^20^ at Keio University Hospital, donor records have been kept rigorously since 1995. It would therefore also be possible to contact past donors and ask their consent to access non‐identifiable or identifiable information.

Concurrently, opinion surveys among past anonymous donors should be conducted to explore their willingness to disclose identifiable and/or non‐identifiable information. If information release is acceptable, the extent to which they wish to have a relationship with the donor‐conceived individual and what expectations they have could also be explored.

Once laws that clarify the status of all parties involved, prospective bans on anonymous donation, and a voluntary register for past donations are established, statistical data and related information can be obtained. Careful monitoring, maintaining a voluntary register, education, and considering the concerns of society and of all parties (ie, donors, donor‐conceived individuals, and parents) indicate an enlightened path forward. This may be effective for removing social stigma and establishing the right to know of donor offspring in a society where donor conception has long been conducted anonymously.

## DISCLOSURES

*Conflict of interest*: Yuri Hibino declared that she has received research grants from JSPS (18KK0340) (collaborative study with Dr Sonia Allan). Sonia Allan declared that she is working with Yuri Hibino with research grants from JSPS (18KK0340). *Human and Animal Rights*: This article does not contain any study with human or animal participants that had been performed by any of the authors.
